# OASIS/CREB3L1 Is Induced by Endoplasmic Reticulum Stress in Human Glioma Cell Lines and Contributes to the Unfolded Protein Response, Extracellular Matrix Production and Cell Migration

**DOI:** 10.1371/journal.pone.0054060

**Published:** 2013-01-15

**Authors:** Ravi N. Vellanki, Liling Zhang, Allen Volchuk

**Affiliations:** 1 Division of Cellular and Molecular Biology, Toronto General Research Institute, University Health Network, Toronto, Ontario, Canada; 2 Department of Physiology, University of Toronto, Toronto, Ontario, Canada; 3 Department of Biochemistry, University of Toronto, Toronto, Ontario Canada; Duke University Medical Center, United States of America

## Abstract

OASIS is a transcription factor similar to ATF6 that is activated by endoplasmic reticulum stress. In this study we investigated the expression of OASIS in human glioma cell lines and the effect of OASIS knock-down on the ER stress response and cell migration. OASIS mRNA was detected in three distinct glioma cell lines (U373, A172 and U87) and expression levels were increased upon treatment with ER stress-inducing compounds in the U373 and U87 lines. OASIS protein, which is glycosylated on Asn-513, was detected in the U373 and U87 glioma lines at low levels in control cells and protein expression was induced by ER stress. Knock-down of OASIS in human glioma cell lines resulted in an attenuated unfolded protein response to ER stress (reduced GRP78/BiP and GRP94 induction) and decreased expression of chondroitin sulfate proteoglycan extracellular matrix proteins, but induction of the collagen gene Col1a1 was unaffected. Cells in which OASIS was knocked-down exhibited altered cell morphology and reduced cell migration. These results suggest that OASIS is important for the ER stress response and maintenance of some extracellular matrix proteins in human glioma cells.

## Introduction

The endoplasmic reticulum (ER) is a vital organelle involved in secretory and membrane protein biosynthesis. When the homeostasis in the ER lumen is perturbed such that an accumulation of unfolded, misfolded or aggregated proteins occurs this creates a state of ER stress. Eukaryotic cells relieve this stress by inducing the unfolded protein response (UPR), which attempts to restore and maintain normal ER homeostasis and function [1]. If the UPR fails to relieve ER stress apoptosis pathways can be initiated [2]. ER stress has been associated with various pathological conditions such as diabetes, atherosclerosis, neurodegenerative disorders, among others [3], [4], [5], [6], [7].

In mammalian cells three principal, ubiquitously expressed ER stress sensors; PKR-like ER kinase (PERK), inositol-requiring enzyme 1α (IRE1α) and activating transcription factor 6 (ATF6) mediate the UPR [8], [9]. Once activated these proteins transduce signals that lead to a transient inhibition in protein translation and transcriptional increases of ER chaperones and degradation components in an attempt to increase protein folding and eliminate misfolded proteins.

In addition to the three main ER stress sensors, additional proteins related to ATF6 such as Old Astrocyte Specifically Induced Substance (OASIS) (also named CREB3L1) are expressed in certain cell types [10], [11], [12]. Similar to ATF6, OASIS is a type II membrane protein with a cytoplasmic N-terminal transcription factor domain and an ER luminal C-terminal domain. OASIS mRNA was first found to be induced in long-term cultured astrocytes and in response to cryo-injury in the mouse cerebral cortex [13]. Subsequent studies found that OASIS mRNA is expressed in a variety of human tissues with predominant expression in pancreas and prostate [14]. More recent studies have shown that OASIS may have a role in differentiation and development of odontoblasts, osteoblasts and pancreatic β-cells [15], [16], [17], [18], [19]. Imaizumi and colleagues were the first to identify that OASIS is an ER stress transducer that translocates from the ER to the Golgi upon ER stress, where it is cleaved by regulated intramembrane proteolysis to release a cytosolic fragment that translocates to nucleus to bind CRE and ERSE (ER stress responsive element) DNA elements [20], [21]. OASIS overexpressed in rat astrocytes up-regulates the expression of GRP78 chaperone, indicating that it may contribute to induction of the UPR [20]. However, OASIS induces the expression of other genes such as extracellular matrix components rather than typical ER stress response genes in osteoblasts [16] and pancreatic β-cells [18].

ER stress has been shown to occur in cancer cells potentially due to the hypoxic conditions experienced by cancer cells *in vivo* [22] and the ER stress response has been suggested to be a potential pathway that can be pharmacologically exploited to induce apoptosis in gliomas [23]. The extracellular matrix has been implicated in cancer cell metastases [24]. For example, in glioblastoma multiforme, the most common brain cancers that are also particularly aggressive [25], the extracellular matrix is involved in cell invasion and migration [26], [27]. Given that OASIS is induced by ER stress and may modulate the extracellular matrix we examined OASIS expression in several human glioma cell lines and the role of this protein in the ER stress response, extracellular matrix production and cell migration.

## Materials and Methods

### Cell Culture

Human glioma cell lines U373, A172 and U87 were obtained from Dr. James Rutka (The Hospital for Sick Children, Toronto). Details for these established cell lines can be found in the following references [28], [29], [30], [31] and the American Type Culture Collection (ATCC) (U87, HTB-14; A172, CRL-1620). The rat C6 glioma cell line was obtained from the ATCC (CCL-107). The cells were cultured and maintained in DMEM (25 mM glucose, 2 mM L-glutamine, 10% FBS, 100 U/ml penicillin, 100 µg/ml streptomycin) at 37°C with 5% CO2.

### RT-PCR and Real-time PCR Analysis

Total RNA was isolated from human glioma and rat C6 cell lines using TRIzol reagent (Invitrogen, Carlsbad, CA) followed by purification using the RNeasy RNA isolation kit (Qiagen, Valencia, CA). cDNA was synthesized using the One step RT-PCR kit (Qiagen) in a PTC-200 (MJ Research, Watertown, MA) thermal cycler. Real-time PCR was performed as described previously [18], [32]. Briefly, total RNA was reverse transcribed to single-stranded cDNA using the High-Capacity cDNA reverse transcription kit (Applied Biosystems). The resulting cDNA was used for real time PCR analysis using the TaqMan Gene Expression system (Applied Biosystems). Primers used were from Applied Biosystems: human OASIS (Hs00369340_m1); human Col1a1 (Hs00164004_m1); human β-actin control (#4333762F).

### Plasmid Generation

Full length rat OASIS cDNA was synthesized from rat pancreatic islet total RNA and subcloned into pCR II Topo vector (Invitrogen) as described earlier [18]. It was then ligated into the expression vector pcDNA 3.1(-) to generate pCMVrOASIS-FL (rOASIS-FL). The human OASIS expression vector (hOASIS-FL) generated as described earlier [18] was subjected to site targeted mutagenesis using the QuickChange kit (Stratagene) to replace asparagines at positions 492 and 513 with alanines, thereby generating glycosylation mutant constructs (OASIS-492ψ, OASIS-513ψ). The constructs were transfected into human glioma cell lines using Lipofectamine 2000 (Invitrogen).

### Knockdown of OASIS by siRNA

Small interfering RNAs (siRNAs) consisting of synthetic annealed RNA duplexes to human OASIS were obtained from Invitrogen, Inc. An siRNA directed to green fluorescent protein (GFP) was used as a control. Cells (1×10^5^) were transfected with 100 nM siRNA using Lipofectamine RNAiMAX reagent (Invitrogen) according to the manufacturer’s instructions.

### Wound Healing Assay

To monitor migration rate, U373 cells (0.4×10^6^) were transfected with 100 nM control or OASIS siRNA for 3 days and incubated at 37°C until cells reached 90% confluence to form a monolayer in a 6 well plate. A p200 pipette tip was used to create a uniform scratch of the cell monolayer followed by a wash with PBS. Fresh DMEM medium (25 mM glucose, 2 mM L-glutamine, 10% FBS, 100 U/ml penicillin, 100µg/ml streptomycin) was added and the cells were incubated for 24–48 h. Representative DIC images of wound healing were monitored with Olympus fluorescence inverted microscope (IX71). Wound closure was determined by quantifying the scratch area using ImageJ v1.42l analysis software.

### Western Blot Analysis

Cells were treated as described in the figure legends and washed with PBS prior to lysis in: (1% Triton X-100, 20 mM HEPES, pH 7.4, 100 mM KCl, 2 mM EDTA, 1 mM PMSF, 10 µg/ml leupeptin, and 10 µg/ml aprotinin, 10 mM NaF, 2 mM Na3VO4, and 10 nM okadaic acid) for 15–20 min on ice. The lysate was centrifuged (10 min) and protein concentration measured using the BCA protein assay kit (Pierce, Inc., Rockford, IL). Equivalent protein amounts were resolved using 10% SDS-PAGE and electro-transferred to Hybond nitrocellulose membranes (GE Healthcare, Piscataway, NJ). Immunodetection was performed with the following primary antibodies: rabbit anti-OASIS (Protein Tech Group, Inc., Chicago, IL), mouse anti-KDEL, mouse anti-PDI (Stressgen Bioreagents, Victoria, BC), rabbit anti-cleaved caspase 3 (Cell Signaling), anti-γ-tubulin (Sigma-Aldrich, St. Louis, MO). The secondary antibodies, anti-mouse HRP (GE Healthcare) and anti-rabbit HRP (Cell Signaling Technology) were used as required and detected by ECL kit (GE Healthcare, RPN2106). Immunoblots were scanned and protein intensities were quantified using Scion Image software (Frederick, MD).

### Immunocytochemistry and Microscopy

Cells were treated as described in the figure legends then fixed and processed for immunofluorescence as described in reference [18]. The primary antibody used was mouse anti-chondroitin sulphate proteoglycan Cat-316 (Abcam, Cambridge, MA; ab78689). Bright-field illumination and fluorescence microscopy were performed with an Olympus fluorescence inverted microscope (IX71) at 60X, NA 0.95 objective. Images were acquired using a CCD camera and processed using Q capture imaging software (Q imaging, Surrey, BC).

### Statistical Analysis

Where applicable results are presented as mean ±SEM. Statistical significance was assessed using the Student’s t-test (two tailed, assuming equal variance) or ANOVA followed by Tukey post-hoc test as indicated in the figure legends (p<0.05 was considered significant).

## Results

### OASIS mRNA and Protein is Induced in Some Human Glioma Cell Lines in Response to ER Stress

**Figure 1:**
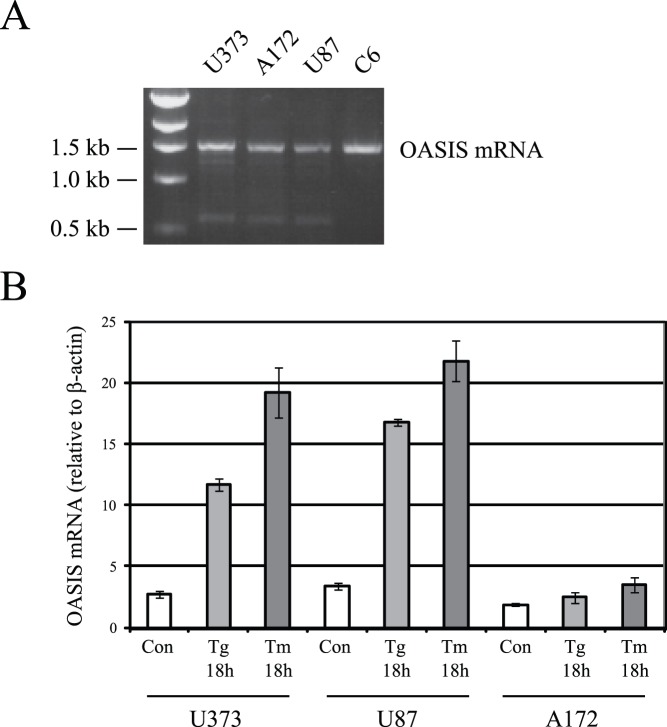
OASIS mRNA is expressed in human glioma cell lines. (A) RNA was isolated lines human glioma cell lines U373, A172, U87 and rat C6 glioma cell lines and OASIS cDNA was amplified by RT-PCR. An ∼1.5 kbp OASIS cDNA was amplified in all cell lines. (B) Human glioma cell lines U373, A172, U87 were treated or not with thapsigargin (TG, 1 µM 18 h) or tunicamycin (TM, 2 µg/ml 18 h). Real time PCR analysis of OASIS mRNA expression relative to cellular β-actin mRNA. Result is from N = 3 independent experiments for each cell line. Bars are SEM.

We investigated OASIS expression in three human glioma cell lines, U373, A172 and U87. The presence of OASIS mRNA in these cell lines was detected by RT-PCR. An ∼1.5 kbp OASIS cDNA was amplified in all three cell lines and in the rat C6 glioma cell line used as a positive control (Figure 1A). By real-time PCR analysis, ER stress-induced by tunicamycin (TM) or thapigargin (TG) resulted in a large increase in OASIS mRNA expression in the U373 and U87 lines, but not in the A172 line (Figure 1B).

**Figure 2:**
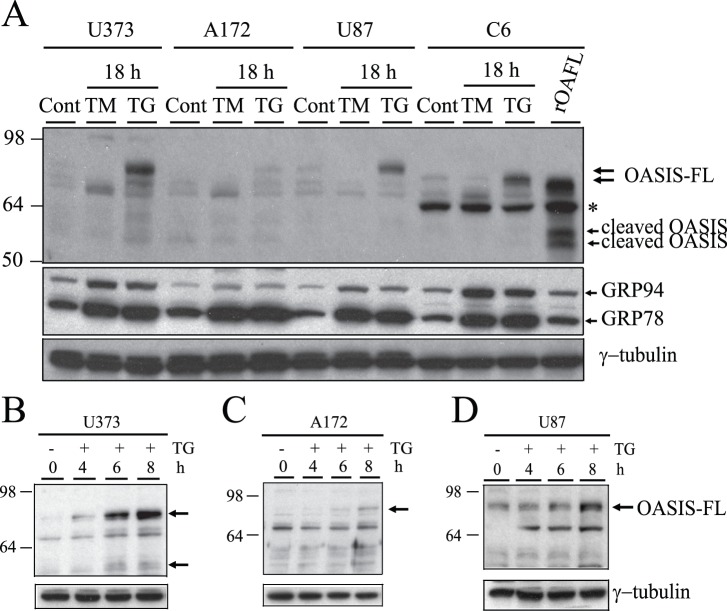
OASIS is a glycoprotein protein induced in some human glioma cells in response to ER stress. (A) Glioma cell lines (U373, A172, U87) and rat C6 cells were treated or not with tunicamycin (TM, 2 µg/ml 18 h) or thapsigargin (TG, 1 µM 18 h), lysed and proteins were resolved by SDS-PAGE and immunoblotted with anti-OASIS, anti-KDEL and anti-γ-tubulin antibodies. Note the higher molecular size of full-length human OASIS compared to rat OASIS protein. A non-specific protein reactive with the OASIS antibody is observed in the rat C6 samples (asterisk). (B-D) Thapsigargin (TG, 1 µM) time course study (0–8 h) for U373 (B), A172(C) and U87 (D) was performed. Note the induction of full-length OASIS protein in U373 and U87 cells, but negligible induction in A172 cells. Appearance of the ∼50 kDa cleaved form of OASIS in response to TG treatment is observed in U373 cells (B, lower arrow). Results are representative of three independent experiments.

To examine OASIS protein expression, the human glioma cell lines were treated or not with tunicamycin (TM) or thapigargin (TG) and cell lysates were prepared. Rat C6 glioma cells transfected or not with rat OASIS were used for comparison. Immunoblot analysis of the cell lysates with anti-OASIS antibody showed barely detectable levels of the ∼85 kDa OASIS protein in all three cell lines under control conditions (Figure 2A, top arrows). The OASIS protein migrates at a higher molecular weight in the human glial cells than in rat C6 cells, which might be due to a differential glycosylation of the human protein. Treatment with TG caused a marked increase in the levels of OASIS protein in U373 and U87 cells and only a minor change in the A172 cell line (Figure 2A). With TM an increase in a lower migrating band was detected in all cell lines, which is likely the unglycosylated form of OASIS (TM is an N-linked glycosylation inhibitor and OASIS is a glycoprotein). Although an increase in the full-length OASIS protein in response to ER stress was detected as has been observed by others [20], ER stress-induced cleavage of OASIS was not easily observed. However, a band migrating at the expected MW for cleaved OASIS was detected in TG treated U373 cells, which have the highest level of OASIS protein expression (Figure 2A and B). The difficulty in detecting cleaved OASIS may be due to nuclear localization of cleaved OASIS and low levels of the cleaved form. We also observed that the ER chaperones GRP78 and GRP94 are markedly elevated in response to ER stress induced by both TM and TG, indicating these human glioma cell lines mount a robust unfolded protein response to ER stress (Figure 2A, middle panel). A time course study from 0–8 h indicated that in U373 and U87 cells full-length OASIS protein was markedly induced by 6 to 8 h of TG treatment, while minimal induction of OASIS was observed in A172 cells (Figure 2B–D). Cleaved OASIS was also detected in response to TG treatment in the U373 cells (Figure 2B, lower arrow).

### Human OASIS is Glycosylated at Aspargine Residue 513

**Figure 3:**
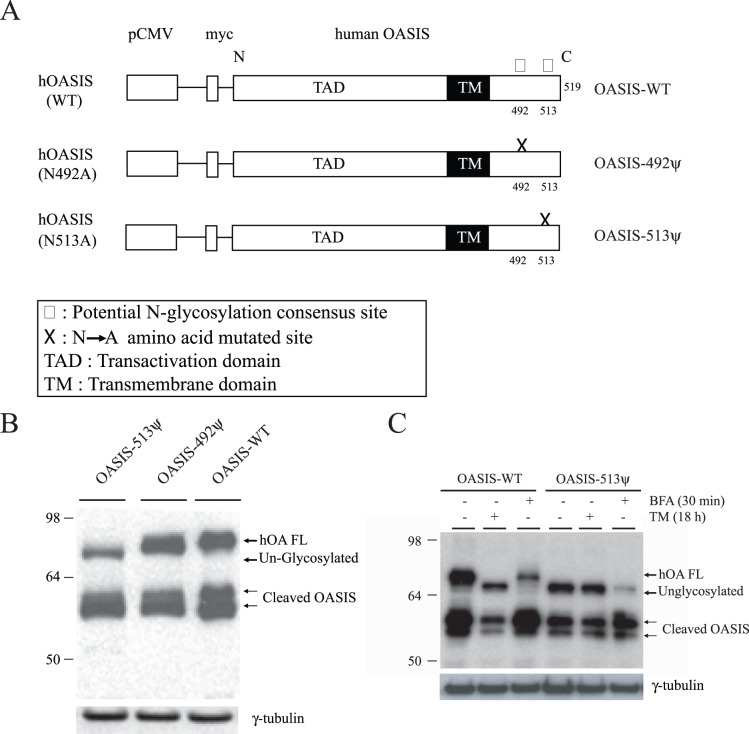
Analysis of human OASIS glycosylation in U373 astrocytes. (A) Potential OASIS glycosylation sites and mutants are indicated. (B) Wild type human OASIS-FL (OASIS-WT) and mutant (ψ)- constructs were transfected in U373 cells and 24 h post transfection were lysed in 1% Triton X-100 lysis buffer and immunoblotted for OASIS (anti-myc) and γ-tubulin (loading control). (C) U373 cells were transfected with either wild-type full-length human OASIS (OASIS-WT) or glycosylation-defective mutant (N-A substitution in residue 513; OASIS-513ψ). The cells were then treated or not with TM or brefeldin A (BFA, 5 µM) as indicated, lysed and immunoblotted for the indicated proteins. Note the complete absence of the ∼80 kDa glycosylated OASIS in cells expressing the mutant protein. Results are representative of three independent experiments.

Mouse OASIS has previously been shown to be glycosylated [20]. Human OASIS has two asparagine residues in the C-terminal ER luminal domain that are potential sites for N-linked glycosylation (Figure 3A). To examine human OASIS glycosylation constructs with asparagine to alanine substitutions at aa492 and aa513 were generated and transfected into U373 cells (Figure 3A–C). Mutation at asparagine (513) completely abolished the ∼80 kDa glycosylated form (Figure 3B, C), while the 492 mutant did not have any significant effect (Figure 3B). Treatment of transfected cells with TM reduced the ∼80 kDa WT protein to ∼70 kDa, which is comparable to the size of the glycosylation mutant OASIS-513. Exposure of WT and transfected cells to brefeldin A (BFA), which causes retrograde transport of protease proteins from the Golgi to the ER, caused a reduction in both glycosylated and unglyosylated forms of OASIS and increased accumulation of the cleaved forms of the protein (Figure 3C).

### OASIS is Required for Maximal Induction of the UPR, Chondroitin Sulfate Proteoglycan Expression and Glioma Cell Migration

**Figure 4:**
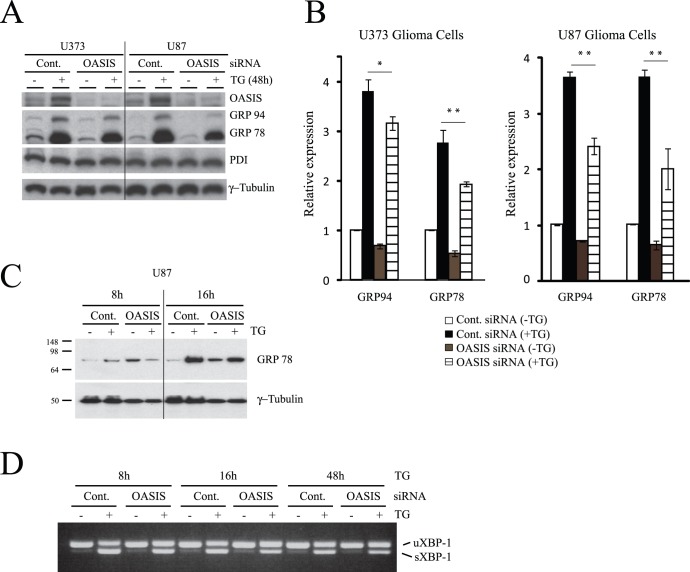
OASIS knockdown attenuates the unfolded protein response to ER stress. (A) Human glioma cell lines were transfected with OASIS siRNA (100 nM) or GFP control siRNA for 7 days. The cells were then treated or not with thapsigargin (TG, 1 µM) for 48 h as indicated, lysed and immunoblotted for the indicated proteins. (B) GRP78 and GRP94 expression was quantified by gel densitometry from 3 independent experiments; * p<0.05 (OASIS siRNA vs. control siRNA), **p<0.01 (OASIS siRNA vs. control siRNA); ANOVA followed by Tukey post hoc test. (C) U87 cells were treated with control or OASIS siRNAs as in (A) then treated or not with TG for the times indicated. Representative immunoblot from N = 3 independent experiments. (D) U87 cells were treated as in (C) then total RNA was isolated and the levels of spliced and unspliced XBP-1 were monitored by RT-PCR. Results is representative of N = 3 experiments.

To address whether endogenous OASIS expressed in human glioma cell lines plays a role in the ER stress response and in extracellular matrix production, we knocked-down OASIS expression using siRNA. As shown in Figure 4A, OASIS siRNA treatment efficiently knocked-down protein expression in both U373 and U87 cells. We examined the ER stress response as measured by the induction of GRP78 and GRP94 in response to TG treatment. Interestingly, the TG induced increase in GRP78 and GRP94 chaperones was blunted in the knock-down cells compared to control (Figure 4A and B). This effect was also observed with shorter TG exposure times (Figure 4C). Analysis of spliced XBP-1 mRNA (indicative of IRE1 activation) showed that the levels of spliced XBP-1 in response to TG-induced ER stress were not affected by OASIS knock-down. Interestingly, spliced XBP-1 was also detected in U87 glioma cells in the absence of TG treatment (Figure 4D), indicating that these fast dividing cells may experience basal ER stress and activation of a mild UPR.

**Figure 5:**
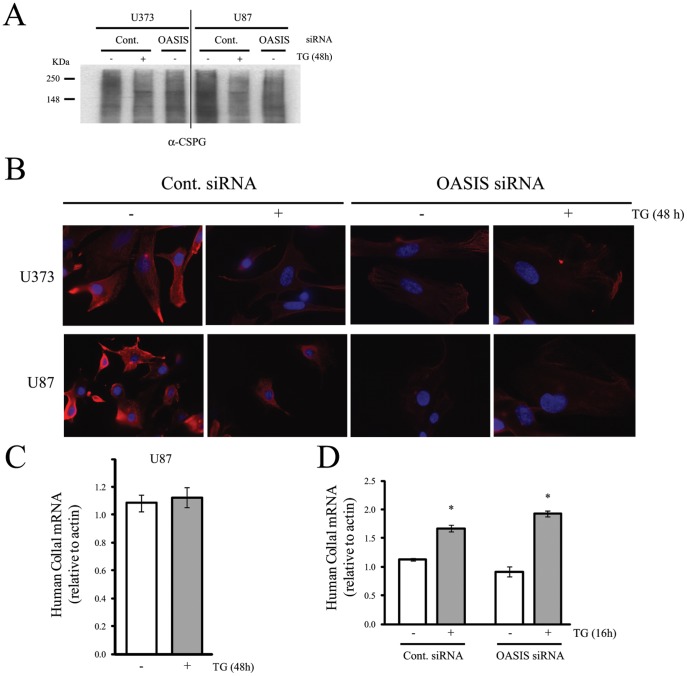
OASIS knockdown reduces chondriotin sulfphate proteoglycan protein expression, but has no effect on Col1a1 gene expression. U373 or U87 cells were treated with control (GFP) or OASIS siRNAs as in Figure 4A. The cells were lysed and immunobotted with an antibody to chondroitin sulfate proteoglycan (A) or were fixed and immunostained with an antibody to chondroitin sulfate proteoglycan and nuclei were stained with DAPI (B). (C) U87 cells were treated or not with TG for 48 h, total RNA was isolated and levels of Col1a1 mRNA were measured by real time PCR. N = 3 experiments. (D) U87 cells were treated with control or OASIS siRNAs as in Figure 4A, then with or without TG for 16 h and levels of Col1a1 mRNA measured by real time PCR. N = 3 independent experiments (Bars are SEM; *p<0.05; TG vs without TG, t-test).

OASIS has also been implicated in modulating extracellular matrix components including chondroitin sulfate proteoglycans [16], [18] and ER stress has been shown to upregulate chondroitin sulfate levels [33]. We thus examined the effect of OASIS knock-down on chondrotin sulfate proteoglycan protein levels using an antibody that recognizes the chondrotin sulfate glycosaminoglycans by western blot and immunofluorescence analysis [34]. ER stress induced by 48 h TG treatment resulted in reduced expression of cellular CSPGs as observed by the reduced high molecular smear detected by the anti-CSPG antibody (Figure 5A) [34]. This was more easily observed by immunofluorescence microscopy, where the CSPG staining was lower in TG treated cells (Figure 5B). Interestingly, OASIS knock-down also effectively reduced chondroitin sulfate proteoglycan expression in non-stresssed U373 and U87 cells, relative to control siRNA treated cells (Figure 5A,B).

Another extracellular matrix component shown to be induced by OASIS in bone osteoblast cells is the collagen gene Col1a1 [16]. Col1a1 mRNA was induced by 16 h, but not by 48 h TG treatment (Figure 5C,D). However, induction of this gene was not affected by OASIS knock-down in U87 glioma cells (Figure 5D).

**Figure 6:**
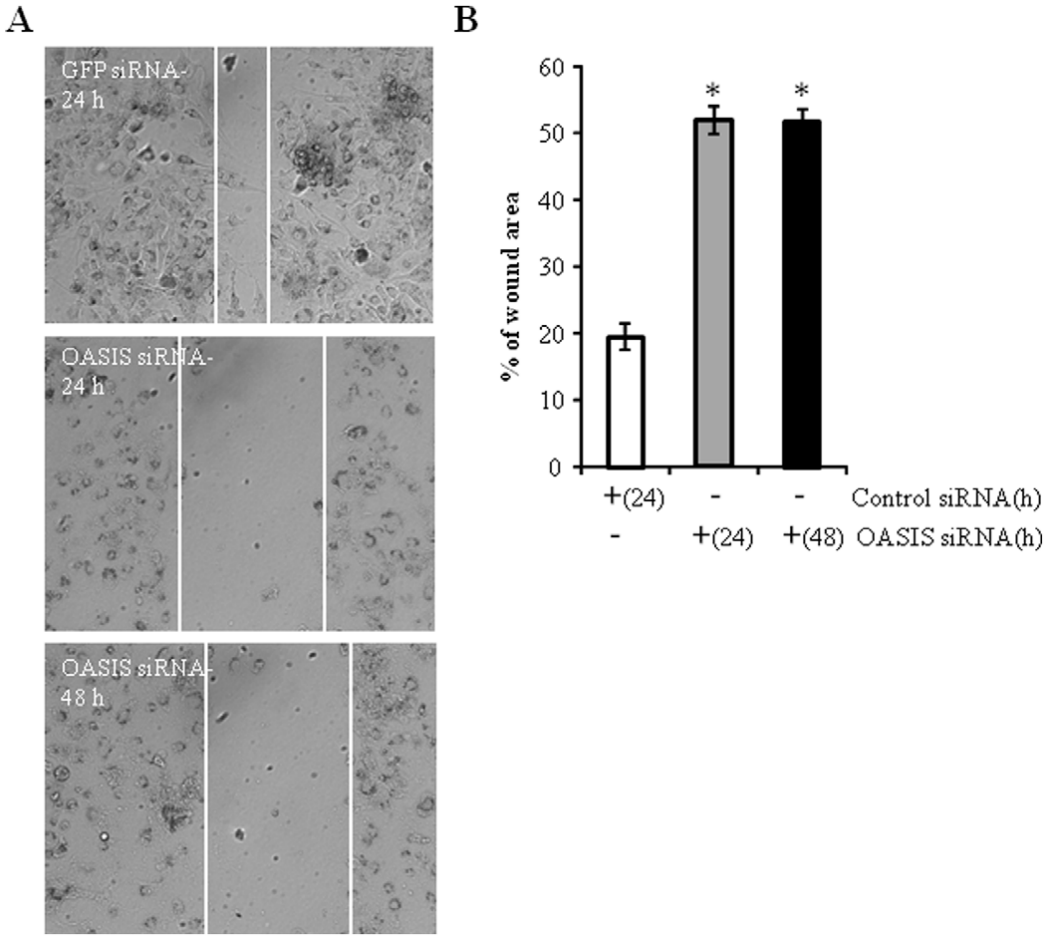
OASIS knock-down perturbs U373 cell migration. (A) U373 cells transfected with 100 nM control (GFP) or OASIS siRNAs for 72 h before scratching the 90% confluent cell monolayers. The cells were then incubated for the indicated times and DIC images were obtained. The approximate cell migration is indicated by the white lines. The images were analyzed by ImageJ and the % wound area was quantified (B) (*p<0.05; OASIS siRNA vs. control siRNA, n = 3). Note the almost complete healing of the wound in control cells and poor migration of cells in which OASIS was knocked-down.

Glioma tumor cells are characterized by their highly invasive and infiltrative capacity. Given that OASIS knock-down resulted in reduced chondrotin sulfate proteoglycan protein expression we examined the migration rate of glioma cells using a wound scratch assay. U373 cells were transfected with control or OASIS siRNAs then a scratch wound was made to the cells and the area was monitored by DIC microscopy. Cells in which OASIS was knocked-down had reduced migration rate compared to control siRNA transfected cells (Figure 6). Whereas the wound area was almost completely colonized after 24 h post-scratch, there was limited migration even after 48 h in the OASIS siRNA transfected cells.

**Figure 7:**
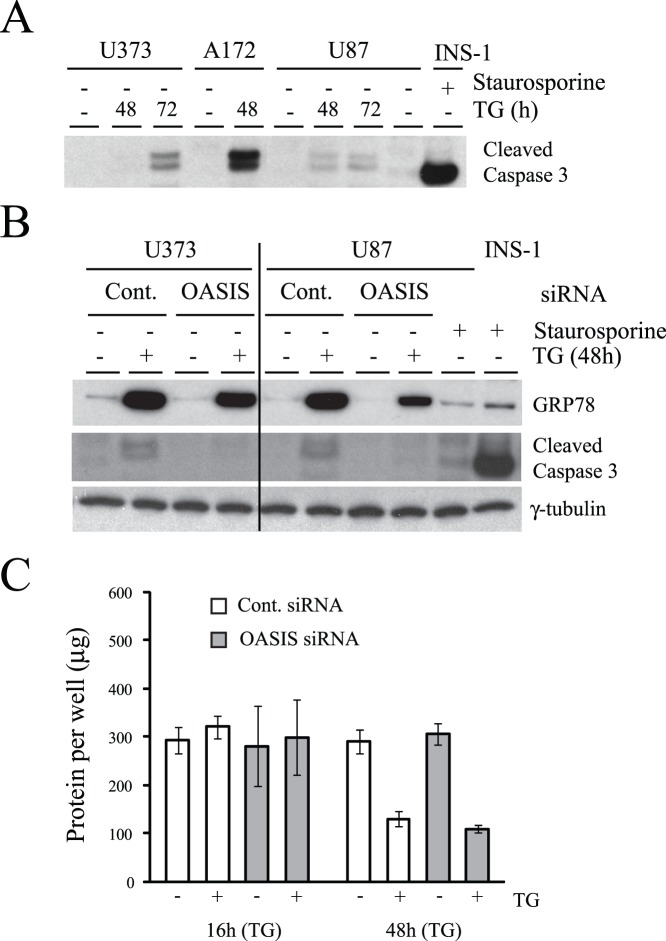
OASIS knock-down does not induce U373 cell apoptosis. (A) U373, A172 and U87 cells were treated with 1 µM TG for the times indicated prior to lysis and immunoblotting for cleaved caspase 3. Rat pancreatic INS-1 insulinoma cells treated with staurosporine were used as a positive control for detection of cleaved caspase 3. (B) U373 cells transfected with 100 nM control (GFP) or OASIS siRNAs for 7 days, then treated or not with 1 µM TG for an additional 48 h. The cells were lysed and immunoblotted for the indicated proteins. Pancreatic INS-1 cells treated with staurosporine (ST, 3 h or 24 h) were used as a positive control for detection of cleaved caspase 3. (C) U87 cells were treated with siRNAs as in (B), then with TG for 16 or 48 h and total protein per well was measured in cell lysates. N = 3 independent experiments. Bars are SEM.

Decreased cell migration could result from reduced cellular growth (proliferation) or increased cell death resulting from apoptosis. We thus monitored cellular apoptosis in control and OASIS siRNA treated cells in the presence and absence of TG-induced ER stress. U373 and U87 human glioma lines were relatively resistant to apoptosis induced by TG requiring 48–72 h of treatment to detect cleaved capsase 3 (Figure 7A). However, caspase 3 was not detected in OASIS or control siRNA transfected cells and OASIS knock-down did not predispose the cells to TG-induced apoptosis (Figure 7B). Thus, OASIS knock-down does not induce significant apoptosis, nor did it affect general cell growth as detected by protein recovery following control or OASIS siRNA treatment (Figure 7C).

## Discussion

OASIS was first identified in mouse astrocytes and glioma cell lines and discovered to be an ER stress response protein [11], [13], [20]. In this study we sought to compare OASIS protein expression and activation in response to ER stress in several human glioma cell lines and determine if OASIS is involved in the UPR, extracellular matrix production and cell migration. Three human glioma cell lines were examined including the U373, A172 and U87 lines [28]. Although OASIS mRNA was readily detected in all three cell lines, protein expression was detected in U373 and U87 cells, but was low to negligibly expressed in A172 cells (Figure 1 and 2). In the U373 and U87 cell lines ER stress induced by TG or TM significantly increased the levels of OASIS mRNA, full-length OASIS protein and cleaved OASIS. We determined that human OASIS is a glycoprotein that undergoes N-linked glycosylation at Asn-513 and thus the mechanism of OASIS activation in response to ER stress may be similar to ATF6. The glycosylation status of p90ATF6 can serve as a sensor for ER homeostasis, resulting in ATF6 activation to trigger the UPR [35]. Thus, in response to ER stress newly synthesized OASIS may be underglycosylated, which may facilitate its export from the ER and activation by proteolysis in the Golgi.

To examine if OASIS modulates UPR genes such as chaperones we knocked down OASIS expression in the U373 and U87 cell lines. The cells were subsequently treated with TG to induce ER stress. Interestingly, knock-down of OASIS reduced the induction of both GRP78 and GRP94 proteins in response to ER stress. This is consistent with results in the literature that have found that GRP78 is a target gene of OASIS in rat C6 glioma cells [20], [36] and indicates that OASIS contributes to maximal induction of the UPR in human glioma cell lines. Our results also suggest that GRP94 may be an OASIS target gene in glioma cells. In osteoblasts and pancreatic β-cell lines, however, OASIS does not induce GRP78 expression [16], [18]. Rather in these cell types OASIS induces expression of genes involved in extracellular matrix production and protein transport, among others.

Rat C6 glioma cells exposed to TG had increased levels of chondroitin sulfate [33]. However, TG treatment of U373 and U87 cell lines resulted in reduced cellular chondroitin sulfate proteoglycan expression. The reason for this is reduction is unclear, although it may relate to the concentration of TG used and length of treatment. Importantly, however, we observed that even in the absence of chemically-induced ER stress, the levels of cellular chondroitin sulfate proteoglycans were reduced in U373 and U87 cells treated with OASIS siRNA. Thus, OASIS may be responsible for maintaining chondroitin sulfate proteoglycan expression even under control conditions in glioma cell lines that express this protein. This is possible given that glioma cells exhibit mild activation of the UPR even under basal conditions (Figure 4D). Another extracellular matrix component shown to be regulated by OASIS in osteoblast cells is the collagen gene Col1a1 [16]. Interestingly, the induction of this gene by ER stress was not affected by OASIS knock-down, suggesting that OASIS is not required for Col1a1 induction in glioma cells or that perhaps another ATF6 family isoform is able to compensate for OASIS loss allowing for Col1a1 induction in glioma cells.

The *in vitro* migration assay identified that OASIS silenced cells have poor migration efficiency. The exact mechanism of this effect is unknown, although presumably OASIS is required for maintaining ECM components that are required for efficient cell migration. Gliomas are characterized by aggressive growth and invasiveness, which is closely related to cell-ECM interactions [23], [25], [26], [27], [37], [38], [39]. Given that OASIS can be induced by ER stress and allows the cell to modulate its matrix we hypothesize that OASIS is likely to be beneficial under chronic, but low intensity ER stress, such as may occur under hypoxic conditions *in vivo* [22]. This will allow the cell to mount a more efficient UPR response and maintain extracellular matrix production, which may contribute to metastasis and cell survival.

Analysis of The Cancer Genome Atlas (cancergenome.nih.gov/) glioma expression database [40], as well as the GBMBase (http://www.gbmbase.org) which focuses on glioblastoma multiforme research, indicates that OASIS and various ER stress response genes are changed in gliomas relative to control tissue (Supplemental data Table S1 and Figure S1). Although OASIS expression can be both increased and decreased in primary human tumors its expression is increased in the majority of glioblastoma subcutaneous xenograph tumors (Supplemental data Table S1 and Figure S1). In future studies it will be important to examine OASIS protein expression and activation in primary human gliomas, as well as examining if targeting ER stress and OASIS may present new strategies to reduce glioma cell growth or infiltration using *in vivo* models.

In summary, we identified that the ER stress sensor OASIS is a glycoprotein that is differentially expressed in human glioma cell lines. OASIS protein is induced by ER stress and appears to contribute to both maximal induction of the UPR (chaperone capacity), as well as maintaining extracellular matrix (CSPG) protein expression in glioma lines that express this protein. Because of these effects, we hypothesize that gliomas that express OASIS may be better off under hypoxic conditions *in vivo* and this may contribute to more resistant and invasive cancers.

## Supporting Information

Figure S1 Expression of selected ER stress and ECM genes in human glioblastoma multiforme (GBM) tumors
(DOCX)

Table S1 Expression of selected ER stress and ECM genes in human glioblastoma multiforme (GBM) tumors
(DOCX)
