# Supplementary material for: OASIS/CREB3L1 Is Induced by Endoplasmic Reticulum Stress in Human Glioma Cell Lines and Contributes to the Unfolded Protein Response, Extracellular Matrix Production and Cell Migration
Source: PLoS One. 2013 Jan 15;8(1):e54060. doi: 10.1371/journal.pone.0054060 (PMC3545929; doi:10.1371/journal.pone.0054060)
Supplement: Figure S1 — Expression of selected ER stress and ECM genes in human glioblastoma multiforme (GBM) tumors (DOCX) [file pone.0054060.s001.docx]

**Supplemental Information:**

**Figure S1.** **Expression of selected ER stress and ECM genes in human glioblastoma multiforme (GBM) tumors**

**A. ER stress response gene expression**


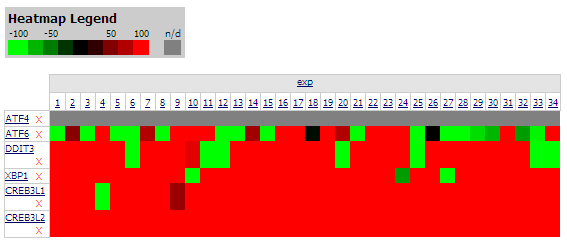


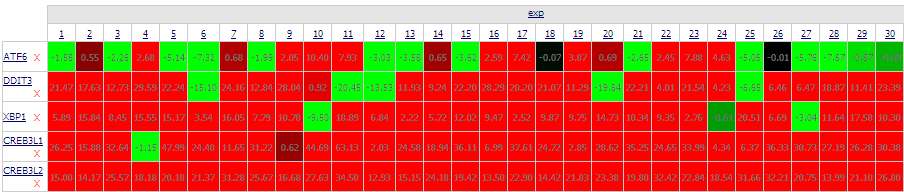

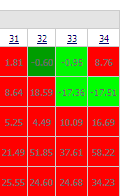


**B. Extracellular matrix (ECM) gene expression**


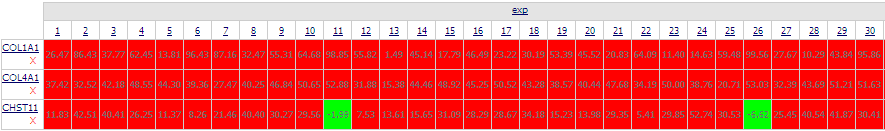

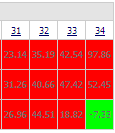


The gene expression data was obtained from **GBMBase that** focuses on glioblastoma multiforme (GBM) research (**http://gbmbase.org**). Hodgson JG, et al. 2009. [Comparative analyses of mRNA expression in glioblastoma multiforme tumors and xenografts.](http://www.ncbi.nlm.nih.gov/pubmed/19139420). Neuro Oncol. 2009;11(5):477-87.

The Microarray (Affymetrix array) viewer (with numeric value) shows transcript expression data from glioblastoma subcutaneous xenografts (#1-34) and non-neoplastic control brain. **Red**-Increased; **Green**-decreased expression. Each row is a gene; each column is a glioblastoma multiforme xenograft tumor. Details regarding experimental conditions and tumor samples can be found at the website (http://gbmbase.org).
