# Supplementary material for: OASIS/CREB3L1 Is Induced by Endoplasmic Reticulum Stress in Human Glioma Cell Lines and Contributes to the Unfolded Protein Response, Extracellular Matrix Production and Cell Migration
Source: PLoS One. 2013 Jan 15;8(1):e54060. doi: 10.1371/journal.pone.0054060 (PMC3545929; doi:10.1371/journal.pone.0054060)
Supplement: Table S1 — Expression of selected ER stress and ECM genes in human glioblastoma multiforme (GBM) tumors (DOCX) [file pone.0054060.s002.docx]

**Supplemental Information:**

**Table S1. Expression of selected ER stress and ECM genes in human glioblastoma multiforme (GBM) tumors.**

| **Gene** |  | **%** |  | **% Increased** | | **% Decreased** |
| --- | --- | --- | --- | --- | --- | --- |
| OASIS/CREB3L1 |  | 47 (199/424) | | 27 |  | 73 |
| CREB3L2 |  | 84 (355/424) | | 99 |  | 1 |
| ATF6 |  | 47 (200/424) | | 98.5 |  | 1.5 |
| DDIT3/CHOP | | 67 (286/424) | | 92 |  | 8 |
| XBP-1 |  | 70 (297/424) | | 98.6 |  | 1.3 |
| COL1A1 |  | 95 (403/424) | | 99.5 |  | 0.5 |
| CHST11 |  | 75 (318/424) | | 97.5 |  | 2.5 |

The gene expression data was obtained using the Data Browser from the human glioblastoma multiforme (GBM) comprehensive genome characterization (The Cancer Genome Atlas-TCGA; [**https://tcga-data.nci.nih.gov/tcga/**](https://tcga-data.nci.nih.gov/tcga/)) Cancer Genome Atlas Research Network. [Comprehensive genomic characterization defines human glioblastoma genes and core pathways.](http://www.ncbi.nlm.nih.gov/pubmed/18772890) Nature. 2008; 455(7216):1061-8. The % of selected ER stress response and extracellular matrix genes in glioblastoma (GBM) tumor samples compared to normal tissue derived using Agilent G4502A_07 gene expression platform (log2 tumor/normal ratio of <= -0.5 or >= 0.5) (i.e. genes increased or decreased at least 1.5 fold in GBM tumors relative to control).

In the case of OASIS/CREB3L1, gene expression is changed by the above criteria in 46.9% of tumor samples (199/424). Within the 47% of tumors with significant changes OASIS expression is increased in 27% of the tumors and decreased in 73%. For the OASIS homologue CREB3L2 it is significantly changed in 84% of tumor samples (355/424) and upregulated in 99% of those tumors. The details on the biospecimens, derivation of the data and the use of the Cancer Genome Atlas Data Brower please refer to the website (<https://tcga-data.nci.nih.gov/tcga/>).

ATF6, Activating transcription factor 6; CREB3L2- Cyclic [AMP](http://en.wikipedia.org/wiki/Cyclic_adenosine_monophosphate) response element-binding protein 3 like 2; DDIT3/CHOP, DNA damage-inducible transcript 3; XBP1-, X-box binding protein; CHST11, Carbohydrate sulfotransferase 11; COL1A1, Collagen type I, alpha 1.
